# Cognitive Behavioral Therapy Plus a Serious Game as a Complementary Tool for a Patient With Parkinson Disease and Impulse Control Disorder: Case Report

**DOI:** 10.2196/33858

**Published:** 2022-09-09

**Authors:** Teresa Mena-Moreno, Lucero Munguía, Rosario Granero, Ignacio Lucas, Almudena Sánchez-Gómez, Ana Cámara, Yaroslau Compta, Francesc Valldeoriola, Fernando Fernandez-Aranda, Anne Sauvaget, José M Menchón, Susana Jiménez-Murcia

**Affiliations:** 1 Department of Psychiatry Bellvitge University Hospital Hospitalet de Llobregat Spain; 2 Centro de Investigación Biomédica en Red-Fisiopatología de la Obesidad y la Nutrición Instituto de Salud Carlos III Madrid Spain; 3 Instituto de Investigación Biomédica de Bellvitge Hospitalet de Llobregat Spain; 4 Department of Psychobiology and Methodology Autonomous University of Barcelona Barcelona Spain; 5 Parkinson's Disease and Movement Disorders Unit Neurology Service, Hospital Clínic Institut D'Investigacions Biomediques August Pi i Sunyer, Institut de Neurociències Universitat de Barcelona (Maria de Maeztu Excellence Center) Barcelona Spain; 6 Centro de Investigación Biomédica en Red sobre Enfermedades Neurodegenerativas Instituto de Salud Carlos III Madrid Spain; 7 Department of Clinical Sciences School of Medicine and Health Sciences University of Barcelona Barcelona Spain; 8 Movement, Interactions, Performance University of Nantes Nantes France; 9 Centro de Investigación Biomédica en Red de Salud Mental Instituto de Salud Carlos III Madrid Spain

**Keywords:** Parkinson disease, impulse control disorder, hypersexuality, multidisciplinary approach, serious game

## Abstract

**Background:**

Impulse control disorders (ICDs) are commonly developed among patients who take dopamine agonist drugs as a treatment for Parkinson disease (PD). Gambling disorder and hypersexuality are more frequent in male patients with PD, with a prevalence over 4% in dopamine agonists users. Although impulsive-compulsive behaviors are related to antiparkinsonian medication, and even though ICD symptomatology, such as hypersexuality, often subsides when the dopaminergic dose is reduced, sometimes ICD persists in spite of drug adjustment. Consequently, a multidisciplinary approach should be considered to address these comorbidities and to explore new forms of complementary interventions, such as serious games or therapies adapted to PD.

**Objective:**

The aim of this study is to present the case of a patient with ICD (ie, hypersexuality) triggered by dopaminergic medication for PD. A combined intervention was carried out using cognitive behavioral therapy (CBT) for ICD adapted to PD, plus an intervention using a serious game—e-Estesia—whose objective is to improve emotion regulation and impulsivity. The aim of the combination of these interventions was to reduce the harm of the disease.

**Methods:**

After 20 CBT sessions, the patient received the e-Estesia intervention over 15 sessions. Repeated measures, before and after the combined intervention, were administered to assess emotion regulation, general psychopathology, and emotional distress and impulsivity.

**Results:**

After the intervention with CBT techniques and e-Estesia, the patient presented fewer difficulties to regulate emotion, less emotional distress, and lower levels of impulsivity in comparison to before the treatment. Moreover, the frequency and severity of the relapses also decreased.

**Conclusions:**

The combined intervention—CBT and a serious game—showed positive results in terms of treatment outcomes.

## Introduction

### Background

Parkinson disease (PD) is a neurodegenerative disorder characterized by a set of motor disturbances, such as rigidity, slowness, and tremor [1], mainly caused by the loss of over 70% of the dopaminergic neurons in the substantia nigra. PD also leads to nonmotor symptoms, such as hyposmia, sleep disorders, and constipation; cognitive impairment disorders (eg, mild cognitive impairment and dementia) [2]; emotional disorders (eg, anxiety, 60%; apathy, 60%; and depression, 35%) [3-5]; and behavioral symptoms. In this latter regard, there is growing evidence that patients with PD are at risk of developing one or more of the four major impulse control disorders (ICDs) [6]. The Diagnostic and Statistical Manual of Mental Disorders, Fifth Edition, by the American Psychiatric Association [7] defines ICDs as a category of behavioral disorders characterized by recurrent maladaptive disinhibited behavior despite adverse personal and relationship consequences.

Dopaminergic medications, particularly dopamine agonists, are associated with the development of ICDs and are related to impulsive-compulsive behaviors in patients with PD [6,8]. The reported prevalence is 14%, but the estimated prevalence of each subtype of ICD is quite variable, often as a consequence of the variability in diagnostic criteria and assessment [9]. Other types of impulsive-compulsive behaviors include punding and compulsive use of dopamine replacement therapy, also known as dopamine dysregulation syndrome, whose prevalence might be over 25% among the idiopathic PD population [10].

The main risk factors for impulse control behavior in patients with PD are young age, the use of dopamine agonists, male gender, drug abuse, depression, smoking, genetic factors, impulsive personality, and family history of ICDs [9,11-20]. In patients with PD, the most frequent forms of ICD include gambling disorder (GD), which is also considered a behavioral addiction, but in any case a disorder related to impulsivity; compulsive shopping; hypersexuality; binge eating disorder, considered as an eating disorder in terms of diagnostics, but highly related to impulsivity as well; and other psychiatric complications (eg, depressive symptoms or sleep disorders) [9,21]. Regarding ICD subtypes, female patients tend to suffer more from compulsive shopping, while hypersexuality is more frequent among male patients [16,22], especially in those with an early onset of PD [16]. Hypersexuality is defined as compulsive sexual behavior, consisting of excessive and distressing sexual thoughts that persist for more than 1 month and interfere with social activity and daily routine [15,16]. It includes excessive preoccupation with sexual thoughts, desire for frequent genital stimulation, internet pornography, promiscuity, and telephone sex, among other behaviors [15,23]. It has been stated that its prevalence is over 4% in users of dopamine agonists and almost 2% in patients with PD who do not take dopamine agonists [22].

ICDs are characterized by a failure to resist a temptation, urge, or impulse that may harm oneself or others. Patients compulsively pursue certain reward-based activities and make poorly informed decisions without foreseeing the potential personal and interpersonal consequences that arise from repetitive participation in these activities. Impulsivity is a fundamental factor in ICD [24,25]. It can be understood as a trait consisting of a stable personality characteristic, which is defined as the tendency to perform behaviors without premeditation and having premature responses to stimuli that often produce adverse consequences. The model by Whiteside and Lynam [26] is one of the most accepted theoretical approaches for defining impulsivity as a multifactorial construct. It was originally formed by the following four dimensions, after which a fifth was added [27]: (1) lack of premeditation, involving acting without thinking; (2) lack of perseverance, representing the tendency to not finish tasks; (3) sensation seeking, encompassing behavior tendencies of trying new and exciting activities or sensations; and (4) positive and negative urgency, including the tendency to act rashly in response to extreme negative or positive emotions. This model has been validated across several age samples, such as children, adolescents, and young adults [28], and was the basis for development of the UPPS-P (Urgency, Premeditation, Perseverance, Sensation Seeking, and Positive Urgency) scale [27,29].

On the other hand, the concept of emotion regulation (ER) is closely related to impulsive behaviors and addictive disorders [30-33]. Different studies have found that many addictive behaviors increased during stressful times, such as smoking and unhealthy eating [34-37] or the use and abuse of alcohol to regulate positive and negative moods [38]. Other research has found that impulsive decision-making may be an attempt to change a negative emotional state [39]. This relationship between impulsive behaviors and emotional state has been studied in previous neuroscience research, indicating that both the prefrontal cortex and the amygdala play key roles in ER [40,41], as well as in impulsive behaviors, reasoning, risk-taking, and decision-making [42-46].

Beyond its relationship with impulsivity, ER is the ability to experience and modulate emotions [47]. ER allows people to access functional resources in stressful situations and allows for the use of appropriate coping strategies [48]. Some of the strategies used to regulate emotions are adaptive (eg, reappraisal), whereas others are maladaptive (eg, suppression) [47,49]. Gratz and Roemer [50] developed the Difficulties in Emotion Regulation Scale (DERS), based on their own theoretical framework that was divided into six dimensions: nonacceptance, goals or directed behaviors, impulse control, emotional awareness, regulation strategies, and emotional clarity. This scale has been amply validated in different age groups [51-54] and different ethnic populations [51,52,55]. 

### Treatment Approaches

The treatment of any of the aforementioned ICDs in PD represents a challenge, due to the fact that not only is the quality of life of patients decreased, but also that of their families or caregivers [56].

Even though impulsive-compulsive behaviors are related to antiparkinsonian medication and ICD symptomatology often subsides when the dopaminergic dose is reduced [57], not all patients will decrease their dosage. Consequently, a multidisciplinary approach should be considered to address these comorbidities [58].

Jiménez-Murcia et al [59] published a protocol for the adaptation of cognitive behavioral therapy (CBT) to treat GD in patients suffering from PD. This therapy consists of 16 sessions and includes specific interventions in PD, such as psychoeducation about specific PD conditions (eg, medication checks and possible side effects) and PD’s relationship with GD, as well as strategies to cope and accept a chronic illness.

Another approach that might be explored is the use of new technologies, considering that they have been shown to be a coadjuvant tool in the rehabilitation of patients with PD [60] and to help in the treatment of ICDs [61,62]. Therefore, the use of new technologies to tackle impulsive-compulsive behaviors in PD is likely to have positive responses. Finally, there are several surgical techniques indicated to reduce the side effects of antiparkinsonian medication [63]. However, the selection of the candidates must be done carefully. Neuropsychological and neuropsychiatric factors also constitute important considerations in predicting the operative outcomes [64], but the risks and benefits of the intervention must be taken into account.

A serious game is understood as “a mental contest, played with a computer [and today, with other electronic devices] in accordance with specific rules, that uses entertainment to further government or corporate training, education, health, public policy, and strategic communication objectives” [65]. In the context of mental health, serious games can be used to complement traditional psychotherapy, providing additional tools for therapists, especially in those patients that exhibit resistance to treatment and might be receptive to new technologies [66,67]. Serious games are becoming widespread resources in personalized medicine approaches, which require that treatment be tailored to the specific needs of patients [68].

A number of studies have reported promising results regarding the efficacy of serious games in treating mental health issues [69]. In particular, individuals experiencing ICDs seem to respond well to interventions aided by serious games [61,62]. In these cases, the gameplay and narrative structure are designed to favor the ER of participants, training them to delay their reactions to external and internal stimuli that usually trigger responses in them.

There is growing interest in using serious games in rehabilitation programs for neurological diseases, although to date, the literature is inconclusive and provides contradictory findings about the efficacy of game-based rehabilitation programs for PD [70]. Some studies indicate that patients with PD can benefit from serious games, as they aim at improving their physical health condition, such as their motor skills, via motion-based video games [60,71] or as they aim at helping in their rehabilitation [72]. Serious games are known to increase the rehabilitation dose by augmenting the intensity and repetition of exercises because of their motivational properties and to improve the follow-up of rehabilitation by the patient [73]. However, games conceived to tackle patients’ mental health, in particular their PD-related ICDs, are also essential. Some authors emphasize the importance of the game design and personalization in order to secure better adherence and a more positive treatment outcome [74].

In addition, new therapy using computer-based ER training or serious video games has emerged [75-81].Tailor-made games that use biosignals to enhance ER have been tested under laboratory conditions. One study reported that individuals increased their emotional awareness and improved their decision-making by refining reward processing through the game’s biofeedback mechanism [82]. Biofeedback can be defined as a technique in which individuals learn to adapt their behaviors based on physiological signals (eg, heart rate variability [HRV]). Thanks to the use of biofeedback, it is possible to connect the emotional reaction to the media display, which helps individuals learn how to regulate their emotions and visually rewards them when doing so appropriately [83]. A great advantage of serious games is that they allow real situations to be recreated in virtual scenarios, while generating a series of cognitive, emotional, and behavioral responses in individuals. This allows for the training of specific skills in a motivating and entertaining way that can be more difficult to achieve with traditional therapies. Self-training via serious games with biofeedback sensors has been shown to reduce general impulsive behaviors and arousal as well as to enhance self-control [61,76]. A serious game called PlayMancer presents these advantages and has shown positive results from training users in relaxation, self-awareness, and self-regulation techniques [79]. Regarding biosignals, HRV responds to autonomic flexibility; therefore, an increased HRV correlates with greater emotional control [84]. Different studies have shown the results of the use of breathing management and biofeedback with measures of HRV to reduce anxiety; reduce psychological stress, as fear of losing control; and make the use of coping strategies more habitual as a form of adaptive ER [85-87].

The aim of this study is to present the case of a patient that suffers from an ICD—hypersexuality—that is triggered by dopaminergic medication for PD. As far as we know, this is the first case study of a patient with PD with a comorbid ICD who was treated with a serious game as a complementary tool of CBT.

## Methods

### Case Description

The patient is a man in his early 30s who was treated at the Gambling Disorder Unit of the Department of Psychiatry at Bellvitge University Hospital, Barcelona, Spain. This is a university hospital belonging to the public health care system and is certified as a highly specialized care center for the treatment of GD and other behavioral addictions. The patient was referred from the Department of Neurology at the Hospital Clinic of Barcelona, Spain.

The patient lives with his parents, both early retirees: the father worked in the audiovisual sector, and the mother worked in a banking institution. The patient has no siblings. He studied vocational training for the position of Higher Technician in Audiovisuals and Shows. He is in a heterosexual relationship of 5 years in length. Regarding recreational activities, he used to go to the cinema and participate in sports (eg, running, football, and the gym) before the PD diagnosis. In general terms, the patient had healthy habits and did not engage in tobacco or alcohol consumption.

### Clinical History

At the age of 30 years, the patient went to the outpatient clinic of the Movement Disorders Unit of the Hospital Clinic of Barcelona, having experienced a few months of predominant bradykinesia and rigidity with a mild rest tremor in his left limbs. After clinical assessment, he was diagnosed with early-onset Parkinson disease in October 2016. He started medical treatment, specifically pramipexol, with good response but with rapid tolerance, leading to a dose increase of up to 3.15 mg per day. Due to high functional requirements, levodopa/carbidopa (Sinemet Plus) was added soon after, reaching doses of 100 mg/25 mg of levodopa/carbidopa four times per day. In December 2017, after 1 year of PD evolution, the patient already presented motor fluctuations and dyskinesias as well as hypersexuality, which led to the withdrawal of the dopamine agonist and the introduction of quetiapine. Intestinal infusion of levodopa gel was eventually administered to try and deliver continuous stimulation as a means of reducing dopaminergic stimulation. Due to the lack of control of his ICD, he was referred to our unit. The patient was evaluated by a clinical psychologist using a clinical interview and the Questionnaire for Impulsive-Compulsive Disorders in Parkinson’s Disease; he was then diagnosed with a nonspecified ICD (ie, hypersexuality) in the context of PD.

The patient also reported visiting websites with pornographic content with the purpose of having telephone sex, as well as frequent visits to prostitutes. The frequency of the dysfunctional behavior was daily during the periods of disease activity and only decreased when the parents exercised strict external control. The patient used to run away from home, if the door was left unlocked, and disappear from his parents’ sight when they went for a walk. He even used to jump out of the window of his home—it was a mezzanine—if his parents were not careful enough. In other words, the impact and interference of this behavior on his life and his family members’ lives were severe.

### Description of the Intervention

After the assessment, the patient started to receive psychological attention. To date, he has received 20 individual sessions of CBT adapted for cases of PD [59]. Different aspects were incorporated into the patient’s treatment in order to treat his hypersexuality problem, taking into consideration his PD. A mood and mental state scan was carried out during the first and second treatment sessions. In these sessions, the patient received psychoeducation about hypersexuality and the therapeutic process and regarding specific PD conditions (eg, medication checks and possible side effects). In sessions 3 and 4, the objectives were to promote gradual abstinence (ie, stimulus control) and explain the importance of the role of cotherapists (ie, his parents). In sessions 5 and 6, difficulties and obstacles related to the treatment were explored. In addition, social skills training and productive time management (ie, planning of hobbies, role playing, and behavioral experiments) were accomplished. In sessions 7 and 8, alternative behaviors to prevent relapse were generated, and the clinical psychologist explained the relationship between cognitions, emotions, and behaviors to the patient. Moreover, specific risk situations and the development of other impulse control behaviors were analyzed, and a medical evaluation was performed in collaboration with a neurologist. From session 9 to 16, the patient performed cognitive restructuring using daily cognitive self-monitoring. Moreover, stimulus control was minimally reduced throughout these sessions, due to the enormous self-control difficulties presented by the patient. Vulnerability factors and similar aspects of thinking were discussed as well. In these sessions, the patient was trained in positive communication styles and new risk behaviors were explored. In the final therapy sessions, several objectives were established: to prevent relapse and conduct future risk factor analysis, to discuss future difficulties and the relevance of maintaining full abstinence, and to analyze the presence of other impulse control behaviors and learn how to cope with somatic chronic disease. Medical evaluation was carried out in collaboration with a neurologist.

The patient attended all the treatment sessions. He was collaborative and fully participated in the sessions. His parents acted as cotherapists throughout the treatment process. Cotherapist duties included learning about hypersexuality and PD, handling risk situations, and helping the patient with his compliance with the treatment guidelines. Furthermore, his parents directly collaborated in some aspects of the treatment, such as stimulus control, and in supporting the patient to find alternative healthy behaviors, such as new distractions and hobbies.

Although a significant improvement was observed in terms of harm reduction with CBT (ie, fewer behaviors related to seeking sexual stimulation and fewer intense and risky consequences), the relapses were still frequent: the maximum time of abstinence from the behavior was 2 weeks. He also showed high levels of impulsivity and deficits of ER, so a complementary treatment was implemented: intervention with a serious game. Thus, the patient received adjunctive treatment with a serious game called e-Estesia. This treatment aimed at improving ER and reducing impulsivity levels through the training of diaphragmatic respiration, which might help reduce physiological activation in stressful situations. e-Estesia is a serious game that integrates the biofeedback mechanism, through heart rate and HRV biosignals, and the diaphragmatic breathing technique to induce a state of well-being in the patient. This combination is possible because cardiac fluctuations are conditioned by the respiratory processes of inspiration and expiration. Heart rate increases during inhalation of air and decreases with exhalation, which is known as respiratory sinus arrhythmia [88,89].

The e-Estesia intervention consisted of 15 sessions, each 10 minutes in length. Some questionnaires were administered on the day of the first session: the DERS; the Emotion Regulation Questionnaire (ERQ); the Symptom Checklist-90-Revised (SCL-90-R), which measures general psychopathology; and the UPPS-P scale, which measures impulsivity. These questionnaires were administered at the end of the 15 sessions.

The first session lasted 30 minutes. The patient was taught how to use the device (ie, an Android tablet), how to place the chest band with the biosensor, and how to use the serious game. He was also taught how to breathe (ie, deeply and slowly) in order to interact with the app. The benefits of this relaxation technique were also explained. The following 14 sessions with e-Estesia took place at the patient’s home, without the supervision of any professional. The patient used the device at home once a day, following the instructions given in the first session. He usually played the serious game around noon, a time that coincided with the on-period of the pharmacological medication.

### The Serious Game e-Estesia

e-Estesia is a serious game that was developed by our research team at Bellvitge University Hospital. It was inspired by PlayMancer, a previous serious game that emerged during a European project at the same hospital, whose application was very successful [61,76,79,90-92]. The PlayMancer app was tested in a significant sample of patients with diagnoses of GD and eating disorders. This evaluation led to the conclusion that a future serious game should be developed using a similar strategy, but with a simpler configuration. It was also apparent to the research team that the app had to be usable at any time and in any situation, allowing the patients to bring the tablet home to self-administer the intervention as they wish. More information about the description of e-Estesia and the usability results can be found in the paper by Mena-Moreno et al [93].

**Figure 1:**
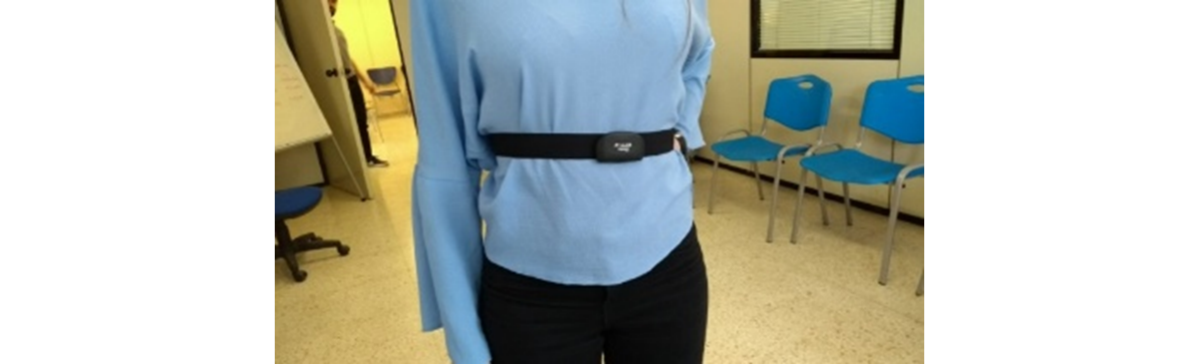
Biosensor placed at the thoracic level to record heart rate variability.

**Figure 2:**
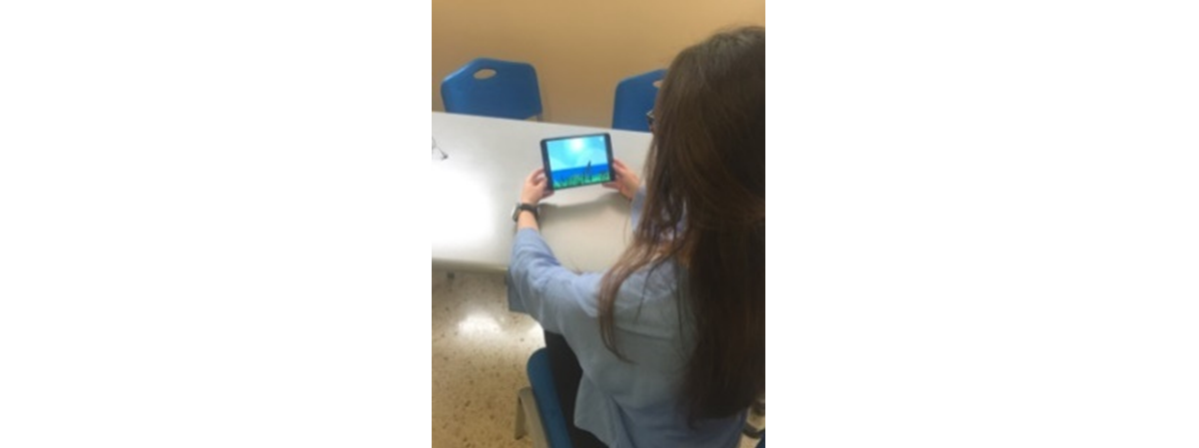
Use of the serious game app e-Estesia.

The serious game e-Estesia is an app that can be used on mobile devices. It consists of a virtual marine landscape that contains a series of animations, from a radiant sun to a tropical storm. The patient must visualize the images while breathing deeply and slowly; the diaphragmatic breathing technique would have been previously taught. A physiological signal that is registered by the app is HRV, which is detected by a biosensor placed at the thoracic level (Figure 1) and is connected through Bluetooth. It allows the patient to interact with the app through the biofeedback technique while he is using the serious game (Figure 2).

**Figure 3:**
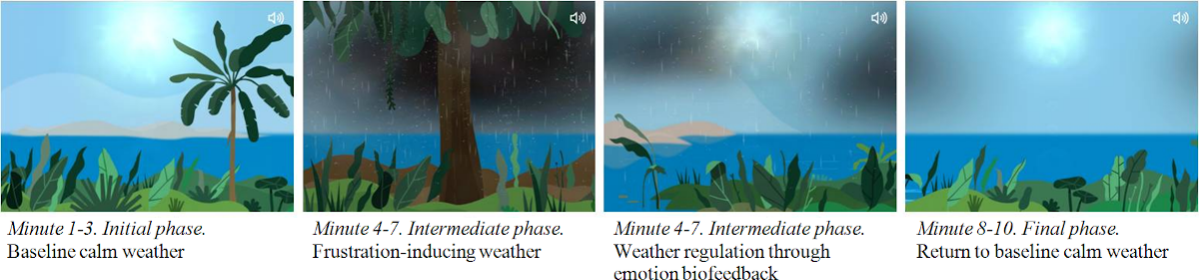
Animation sequence of the serous game app e-Estesia.

Each session with e-Estesia lasts 10 minutes and is divided into three periods of time. During the first and last 3 minutes, the patient should try to relax while visualizing the landscape and breathe deeply. During the middle 4 minutes, gray clouds and rain appear on the screen, simulating an intense storm; if the patient remains relaxed (ie, low activation level) and breathes deeply (ie, diaphragmatic breathing), these clouds will dissipate, which is the reward element (ie, reinforcement of the behavior to be implemented). However, if the patient stops breathing deeply and the situation agitates him or her, the storm will increase in intensity (ie, a signal that the behavior is not appropriate), the rain will be heavier, and the sound of falling water will increase. These 4 minutes allow patients to be trained to face stressful or emotionally negative situations using breathing as a complementary therapeutic strategy. These techniques are the ones learned during their standard treatment (Figure 3).

The patient plays an active role throughout the session with e-Estesia. He or she must manage their breathing (ie, breathing deeply and slowly), and the app reports feedback through the animations that appear on the tablet screen. The patient is in continuous interaction with the serious game, receiving information about whether or not he or she is performing the task adequately, reinforcing learning, while at the same time modifying his or her behavior and physiological responses to achieve the marked objective.

### Ethics Approval

This study was conducted according to the guidelines of the Declaration of Helsinki of 1975 (revised in 2013) and was approved by the Research Ethics Committee of the Bellvitge University Hospital (reference No. PR208/22). Signed informed consent was obtained from the patient involved in the study. Since the patient was of legal age, it was not necessary for his parents to sign the consent form, although they were also informed verbally about the study by the group’s principal investigator. Both the patient and his parents accepted, and the patient himself signed the informed consent document.

## Results

### Physiological Activity Registered in e-Estesia

**Figure 4:**
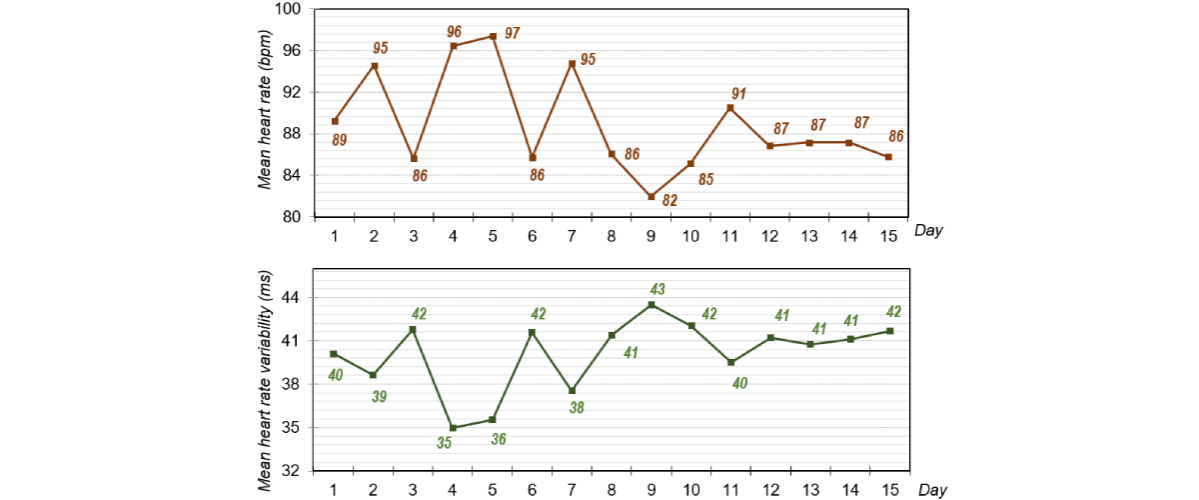
Mean heart rate and heart rate variability over 15 days of using the serious game app e-Estesia.

Figure 4 shows the results of the heart rate and HRV measurements during 15 days of using e-Estesia. These physiological measures show two clearly different periods throughout the treatment with this system: the first half versus the second half of the treatment period. The first period included the first 7 days of practice, while the second period comprised the last 8 days. When comparing the two periods, we found that the mean heart rate decreased from the first period (92 bpm) to the second period (86.38 bpm). Regarding HRV, we also found changes between these two periods. During the first half of the treatment period (mean HRV 38.86 ms), the patient displayed lower HRV than in the second period (mean HRV 41.38 ms).

### Psychopathological and Clinical Indices

**Figure 5:**
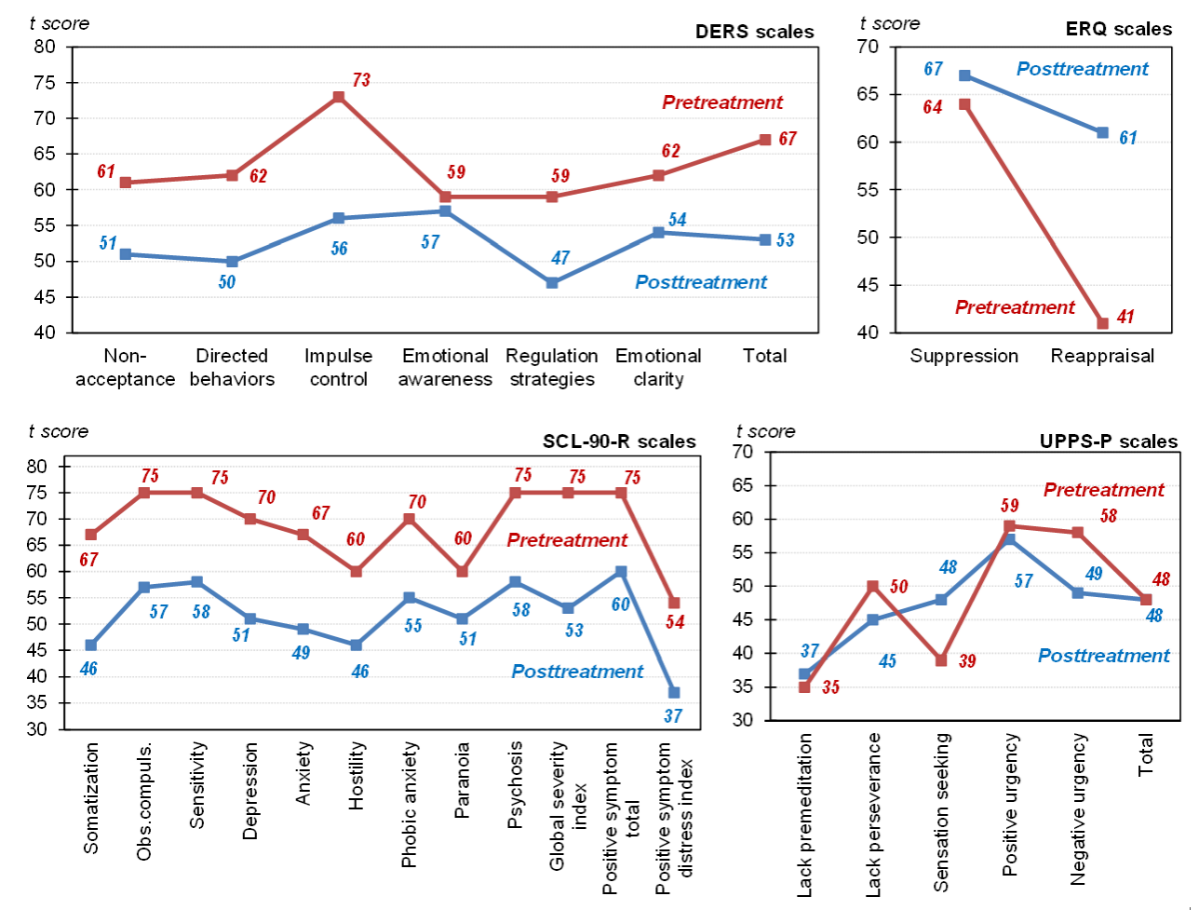
Pre- and posttest changes in the psychometric measures. DERS: Difficulties in Emotion Regulation Scale; ERQ: Emotion Regulation Questionnaire; GSI: gait symmetry index; Obs. compuls.: obsessive compulsion; premeditat.: premeditation; SCL-90-R: Symptom Checklist-90-Revised; UPPS-P: Urgency, Premeditation, Perseverance, Sensation Seeking, and Positive Urgency.

Figure 5 shows the pre- to posttreatment changes in the psychometric scales of the study measuring the dysregulation of emotional state, psychopathology, and impulsivity levels. Decreasing standardized *t* scores from pre- to posttreatment were observed for the DERS: the highest change was observed for impulse control (17 points, from *t*=73 to *t*=56) and the lowest change was observed for emotional awareness (2 points, from *t*=59 to *t*=57). All the standardized *t* scores for the DERS at posttreatment were in the normal range (*t*<60) [94]. Increasing standardized *t* scores were observed from pre- to posttreatment for both strategies measured using the ERQ: reappraisal (20 points, from *t*=41 to *t*=61) and suppression (3 points, from *t*=64 to *t*=67).

For the SCL-90-R scales, decreasing standardized *t* scores from pre- to posttreatment were also observed: the highest change was for global severity index (22 points, from *t*=75 to *t*=53) and the lowest change was for paranoia (19 points, from *t*=60 to *t*=41). All the standardized *t* scores for the SCL-90-R scales at posttreatment were in the normal range (*t*<63) [95].

Regarding the UPPS-P scales, decreasing standardized *t* scores from pre- to posttreatment were also observed for lack of perseverance (5 points, from *t*=50 to *t*=45), positive urgency (2 points, from *t*=59 to *t*=57), and negative urgency (2 points, from *t*=50 to *t*=48). No pre- to posttreatment change was observed for the total score, while lack of premeditation minimally increased (2 points, from *t*=35 to *t*=37) and sensation seeking increased (9 points, from *t*=39 to *t*=48).

## Discussion

### Principal Findings

As far as we know, this is the first case study of a patient with PD with a comorbid ICD treated with a serious game as a complementary tool to CBT. e-Estesia was employed with partial but clinically significant success, with amelioration of the symptoms related to ER and impulsivity. A significant improvement in harm reduction had already been observed with CBT (ie, fewer behaviors related to seeking sexual stimulation, with less intensity and fewer risky consequences).

After the intervention with e-Estesia, as expected, the patient presented a change in the collected psychophysiological measures (ie, heart rate and HRV). Regarding psychophysiological variables, a decrease in heart rate response was seen during the last sessions in comparison with the first ones. Regarding cardiac activity, HRV was found to be higher at the end of the e-Estesia treatment in comparison with the first part of treatment. HRV is a measure that responds to autonomic flexibility and, therefore, an increased HRV correlates with greater emotional control [84]. Previous studies have shown that cardiac activity can be modulated by respiratory training using biofeedback applications [96-98]. Diaphragmatic breathing increases the vagal tone, activating the relaxation response of the parasympathetic nervous system [99]. Hence, the diminished heart rate response after the intervention could point toward an attenuated emotional response due to the treatment with e-Estesia. Similarly, the increase in HRV could be related to the skills learned by the patient when using e-Estesia, which probably improved ER processes. Both variables support the idea of a more adaptive affective response, probably related to better ER processes. Therefore, the treatment with e-Estesia may have produced a diminished emotional response to negative events as well as better affect regulation skills.

The results from the psychophysiological measures correspond to the changes at the clinical level collected by the psychometric measures. After the intervention with e-Estesia, an improvement was observed in general psychopathology, such as better ER and lower scores in some of the impulsivity scales.

Decreased standardized *t* scores for the DERS and the higher use of reappraisal strategies as observed by the ERQ indicated a better ER after the intervention with e-Estesia [49,50]. Reappraisal is a strategy that results in positive emotional and physical responses to emotion-eliciting stimuli, whereas various forms of suppression and avoidance have been implicated in psychopathology [47,49].

After the intervention with the serious game, the patient showed fewer difficulties in ER [50,94]. The impulse control subscale showed the greatest change, reflecting fewer difficulties in controlling impulses and behaviors in the presence of negative emotions. Also, a notable difference in the directed behaviors scale was also observed after the intervention with e-Estesia, indicating fewer difficulties in adopting goal-oriented behaviors and performing a task in the presence of negative emotions [50].

According to the impulsivity measures, there was a decrease in the scores for the positive and negative urgency UPPS-P subscales, which represent emotion-related aspects of impulsivity, with the negative urgency subscale score being even higher. These personality traits are defined as the tendency to act rashly when experiencing extreme emotional states [26,27,100], for which it is possible to hypothesize that this result might be associated with the improvements in ER observed after the intervention; as it has been mentioned in the literature, important associations between impulsivity and ER have been found in addictive disorders [30-33]. Therefore, the use of e-Estesia could be of benefit in the reduction of impulsivity reactions through the training of self-ER. As previous studies have shown, improvements in ER have a positive impact on the reduction of the performance of characteristic impulsive behaviors in other ICDs [101,102].

Another important improvement was found in the lack of perseverance UPPS-P subscale. Lack of perseverance is related to poor concentration on boring or difficult tasks [26,27], and it has been found in patients with PD who have ICD [103]. This result is in concordance with the literature, indicating that the use of serious games could be of benefit to increase attention in order to develop abilities and competencies [104,105]. Therefore, the use of e-Estesia could not only improve ER or impulsivity but other neurocognitive processes as well, such as attention and learning, which seem to be disturbed in PD. However, the level of sensation seeking continues increasing despite the treatment. This result could be related to the typical course of the disease. Higher levels of this impulsivity dimension have been found in patients with PD who have ICD [103,106,107].

Clinical symptoms measured with the SCL-90-R also showed a significant overall reduction, indicating a better psychopathological state and lower distress level at the end of the treatment [108].

Due to the severity and complexity of the case, absolute abstinence was not achieved—although, it was not the goal of the therapy—but the combined intervention (ie, CBT plus the serious game) resulted in a significant improvement in harm reduction, impulsivity, ER, and mood. It also increased the awareness and self-control of the problem behavior. In addition, the impact of this behavior on the patient’s life and environment was reduced.

### Limitations

Although the use of e-Estesia has been effective, due to the complexity involved in dealing with this type of comorbid problem, it is difficult to attribute the success of the intervention to the serious game only, since other factors, such as the effect of the medication, may have come into play regarding the therapeutic outcome.

### Conclusions

In the clinical case presented, different interventions from a multidisciplinary perspective were carried out in order to treat ICD (ie, hypersexuality) triggered by dopaminergic medication for PD. The result of the different interventions has been positive: a combination of a manualized CBT approach plus an intervention with a serious game (e-Estesia).

Due to the different symptoms developed in this type of patient, in which the lack of impulse control itself is encouraged by the treatment (ie, usually dopaminergic medications) [6,8,9,109] for the first medical condition (ie, PD), the need arises to apply a multidisciplinary perspective. Although impulsive-compulsive behaviors are related to antiparkinsonian medication, and even though ICD symptomatology often subsides when the dopaminergic dose is reduced [57], not all patients could decrease the dosage. Hence, there is a need to apply a multidisciplinary approach to address these comorbidities [58].

In this regard, the implications of this first approximation of the use of a serious game as a coadjuvant tool in the treatment of patients with PD who have ICD are important, since positive results were found regarding the reduction of impulsivity and improvements in ER. This first approximation highlights the importance of developing multidisciplinary interventions for the treatment of complex conditions, such as comorbid PD and ICD.

## Abbreviations

CBT: cognitive behavioral therapy
CERCA: Centres de Recerca de Catalunya
CIBERObn: Centro de Investigación Biomédica en Red-Fisiopatología de la Obesidad y la Nutrición
CIBERSAM: Centro de Investigación Biomédica en Red de Salud Mental
DERS: Difficulties in Emotion Regulation Scale
ER: emotion regulation
ERQ: Emotion Regulation Questionnaire
FEDER: Federacion Espanola de Enfermedades Raras
GD: gambling disorder
HRV: heart rate variability
ICD: impulse control disorder
ISCIII: Instituto de Salud Carlos III
PD: Parkinson disease
SCL-90-R: Symptom Checklist-90-Revised
UPPS-P: Urgency, Premeditation, Perseverance, Sensation Seeking, and Positive Urgency
